# Robust Anchor-Aided GNSS/PDR Pedestrian Localization via Factor Graph Optimization for Remote Sighted Assistance

**DOI:** 10.3390/s25175536

**Published:** 2025-09-05

**Authors:** Sen Huang, Jinjing Zhao, Yihan Zhong, Yiding Liu, Shengyong Xu

**Affiliations:** 1 School of Electronics, Peking University, Beijing 100871, China; senhuang@stu.pku.edu.cn (S.H.); zhaojinjing@pku.edu.cn (J.Z.); 2301213327@pku.edu.cn (Y.L.); 2 Department of Aeronautical and Aviation Engineering, The Hong Kong Polytechnic University, Hong Kong, China; yi-han.zhong@connect.polyu.hk

**Keywords:** factor graph optimization (FGO), global navigation satellite system (GNSS), pedestrian dead reckoning (PDR), road-anchor, video-based map, remote sighted assistance (RSA), smartphone

## Abstract

Remote Sighted Assistance (RSA) systems provide visually impaired people (VIPs) with real-time guidance by connecting them with remote sighted agents to facilitate daily travel. However, unfamiliar environments often complicate decision-making for agents and can induce anxiety in VIPs, thereby reducing the effectiveness of the assistance provided. To address this challenge, this paper proposes a video-based map assistance method. By pre-recording pedestrian path videos and aligning them with geographic locations, the system enables route preview and enhances navigation guidance. This study introduces a factor graph optimization (FGO) algorithm that integrates Global Navigation Satellite System (GNSS) and pedestrian dead reckoning (PDR) data for pedestrian positioning. It incorporates road-anchor constraints, a turning-point-based anchor-matching method, and a coarse-to-fine optimization strategy to improve the positioning accuracy. GNSS provides global reference positions, PDR offers precise relative motion constraints through accurate heading estimation, and anchor factors further enhance localization accuracy by leveraging known geometric features. We collected data using a smartphone equipped with a four-camera module and conducted tests in representative urban environments. Experimental results demonstrate that the proposed anchor-aided FGO-GNSS/PDR algorithm achieves robust and accurate positioning, effectively supporting video-based map construction in complex urban settings. With anchor constraints, the mean horizontal positioning error was reduced by 42% to 65% and the maximum error by 38% to 76% across all datasets. In this study, the mean horizontal positioning error was 1.36 m.

## 1. Introduction

Visual impairment is a prevalent global health issue. According to the data from The Lancet Global Health Commission [1], as of 2020, there were 596 million visually impaired people (VIPs) globally, including 43 million who were blind. By 2050, these figures are expected to rise to 895 million and 61 million, respectively. Given that visual information accounts for approximately 80% of human perception, the large population of VIPs faces significant challenges in their daily lives and mobility.

With the rapid advancement of smartphone technology and wireless communication, Remote Sighted Assistance (RSA) systems have emerged to support VIPs in navigation and other daily tasks [2]. A typical RSA system includes four core components: visual information acquisition, data transmission, remote assistance, and user feedback. In such systems, users can request help via a mobile platform, and remote sighted agents provide guidance based on real-time visual data captured from wearable or mobile cameras. RSA applications have been developed for mobility assistance [3], text recognition [3,4,5], and object identification [3,4,5]. Notable commercial platforms include BeMyEyes [3], TapTapSee [4], and Aira [5], while research prototypes include VizWiz [6], BeSpecular [7], and CrowdViz [8]. Despite recent progress, RSA systems still face several critical challenges, including reliable real-time communication, precise localization in GNSS-degraded areas, environmental understanding, and intuitive user feedback design.

**Figure 1:**
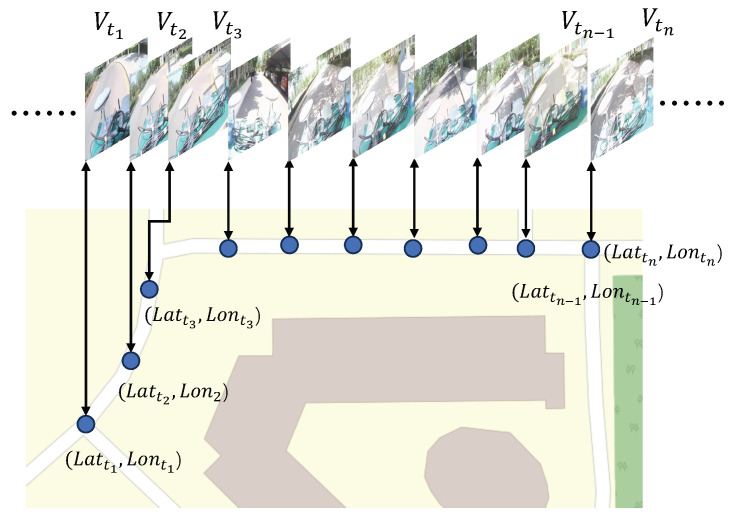
A schematic of the video-based map proposed in this study. Spatial positions are represented using latitude and longitude coordinates, with associations established between video frames and geographic locations. The background map layer is derived from OSM data [12].

Recent studies have identified two key challenges in RSA systems: (1) remote sighted agents experience stress and encounter difficulties in making accurate judgments when navigating unfamiliar environments, and (2) visually impaired users often feel anxious and uneasy when navigating unknown areas [2,9]. Research has also shown that incorporating 3D maps can help remote sighted agents better understand the environment [10]. Therefore, enabling both remote sighted agents and visually impaired users to preview route information in advance has the potential to significantly enhance RSA effectiveness and user experience. Although commercial mapping services such as Google Maps [11] and OpenStreetMap (OSM) [12] provide extensive road network and satellite imagery data, they are not well-aligned with real-world first-person perspectives, thereby limiting their effectiveness for pedestrian navigation. In this study, we use the term “real-world first-person perspectives” to describe video content captured from the viewpoint of a pedestrian walking along the route, closely replicating what a VIP would perceive during navigation. Unlike Google Maps or similar services, which primarily provide top-down maps or static street-view images, this approach aligns with a VIP’s forward-facing, step-by-step experience. Furthermore, publicly available imagery such as Google Street View is often outdated, lacks comprehensive coverage, and may not accurately reflect temporary environmental changes, such as construction or obstacles. Moreover, existing map data primarily focuses on the vehicular road network, resulting in sparse or incomplete coverage of pedestrian pathways. To address this limitation and support the need for prior environmental awareness in RSA services, this study proposes a video-based map, as presented in Figure 1. The system captures pedestrian environments through pre-recorded videos and establishes precise spatiotemporal correspondences to enhance mobility assistance in RSA scenarios. Remote sighted agents can preview the video-based map prior to the assistance session and summarize key information—such as route layout, landmarks, and potential obstacles—to visually impaired users via voice instructions. Compared with costly 3D mapping technologies, our video-based map offers a lightweight, low-cost, and perceptually aligned alternative that is well suited for outdoor remote vision assistance in real-world urban environments.

To precisely associate video frames with geographic locations, spatial positions are represented using latitude and longitude coordinates. While video acquisition is relatively straightforward, the primary challenge lies in establishing an accurate mapping between time and space. Numerous studies have investigated smartphone-based pedestrian localization methods [13,14,15,16,17]. Smartphones equipped with Global Navigation Satellite System (GNSS) modules estimate absolute positions by measuring pseudoranges and applying trilateration techniques [18,19]. However, common urban environments with buildings and trees often cause multipath propagation and Non-Line-of-Sight (NLOS) effects, leading to significant GNSS positioning errors or even failures [20,21]. To improve GNSS accuracy, researchers have developed algorithms based on weighted least squares [22,23], Kalman filtering (KF) [24,25], extended Kalman filtering (EKF) [26], and factor graph optimization (FGO) [27,28]. Among these, FGO is particularly effective, as it leverages a sequence of historical measurements to produce more accurate estimates [29]. However, a study from the Hong Kong Polytechnic University indicates that even FGO based solely on GNSS can result in errors exceeding 20 m in urban settings [30].

In contrast to GNSS, Inertial Measurement Unit (IMU)-based localization provides continuous relative positioning without relying on external signals [31,32,33]. Most modern smartphones are equipped with MEMS-based IMUs, which enable pedestrian dead reckoning (PDR) using accelerometer, gyroscope, and magnetometer data [13,34]. PDR typically involves three key components: step detection, step length estimation, and heading estimation. It provides relative motion estimates between consecutive steps, which are then accumulated to reconstruct the pedestrian trajectory. However, due to sensor noise and drift, PDR is susceptible to cumulative errors that can reach tens of meters [13,34].

To overcome the limitations of individual systems, GNSS/PDR fusion algorithms integrate the absolute positioning accuracy of GNSS with the continuous tracking capability of PDR, enabling robust localization in urban environments [14,15,16,17,35,36,37,38]. Recent efforts have increasingly focused on smartphone-based fusion methods [14,15,16,17,39,40,41]. Angrisano et al. [39] and Basso et al. [41] proposed loosely coupled fusion architectures based on EKF frameworks to integrate GNSS and PDR, thereby improving system reliability. Meng et al. [15] introduced a KF-based GNSS/PDR fusion method with adaptive measurement adjustments. Zhang et al. [40] applied an adaptive EKF (AEKF) to enhance robustness and mitigate cumulative errors. Jiang et al. [14] pioneered the use of FGO for smartphone-based GNSS/PDR fusion, proposing both position and step-length constraint factors. Zhong et al. [16] introduced PDR displacement factors derived from accelerometer data, which were jointly optimized with GNSS pseudorange and Doppler observations, achieving an average positioning accuracy of 18.51 m. Building on this work, Jiang et al. [17] developed an adaptive FGO (A-FGO) framework that dynamically adjusts observation covariances to suppress outliers, achieving sub 4 m accuracy in certain test scenarios. Overall, FGO-based GNSS/PDR fusion methods have demonstrated superior localization accuracy compared to EKF-based approaches [14,17,29,42].

This study proposes a smartphone-based method for video-based map collection, motivated by the low cost and the widespread adoption of smartphones in RSA systems. In addition, we develop a high-precision pedestrian localization algorithm that builds upon existing solutions. For data acquisition, we employ a custom-designed multi-camera module and a mobile application to capture forward-facing video with a 180° field of view. For localization, we adopt an FGO framework that fuses GNSS and PDR data. To enhance performance, we introduce novel road anchor factors, an anchor-matching algorithm, and a coarse-to-fine optimization strategy [43]. In this framework, GNSS factors provide global position constraints to correct accumulated PDR drift; PDR factors enhance robustness by mitigating GNSS outliers; and anchor factors leverage road features to correct residual localization errors. The FGO-based fusion framework further refines trajectories by integrating multi-source historical data. The main contributions of this work are summarized as follows:

1. We propose a method for constructing a video-based map by associating video frames with corresponding spatial locations to enhance the RSA service. This map serves as a preview tool for RSA agents and provides prior environmental knowledge for visually impaired users, which helps to reduce anxiety in unfamiliar environments. It also provides valuable assistance under low-light or poor network connectivity conditions, thereby enhancing the overall safety and reliability of RSA services.
2. To establish accurate spatiotemporal correspondence, we propose a novel FGO-based GNSS/PDR fusion framework. This framework integrates road-anchor constraints, a turning-point-based anchor-matching method, and a coarse-to-fine optimization strategy to improve positioning accuracy. The incorporation of anchor factors further refines the localization results.
3. We conducted extensive real-world experiments and collected datasets from three representative urban routes to evaluate the proposed localization algorithm. The results demonstrate that our anchor-aided FGO-based GNSS/PDR fusion method achieves high localization accuracy that is sufficient for video-based map construction.

The remainder of this paper is organized as follows. Section 2 presents the proposed method for constructing the video-based map, with a focus on the localization framework, including the PDR algorithm, the PDR factor, the anchor factor, the GNSS factor, and the FGO-based data fusion strategy. Section 3 presents the experimental results obtained from two real-world test scenarios and provides a detailed performance analysis. Section 4 discusses the proposed PDR algorithm, the role of anchor factors, system robustness, and potential directions for future research. Finally, Section 5 concludes the paper.

## 2. Materials and Methods

The construction of the proposed video-based map involves two primary stages: data acquisition and post-processing. In the data acquisition phase, participants are equipped with a smartphone and a multi-camera module to capture video data, GNSS positioning measurements, and sensor readings from the IMU and the attitude and heading reference system (AHRS). A major challenge in this system lies in accurately associating the captured video stream with corresponding spatial coordinates, which is the core focus of the present study. During the post-processing stage, the data are used to estimate pedestrian positions and to establish a correspondence between video timestamps and spatial locations.

**Figure 2:**
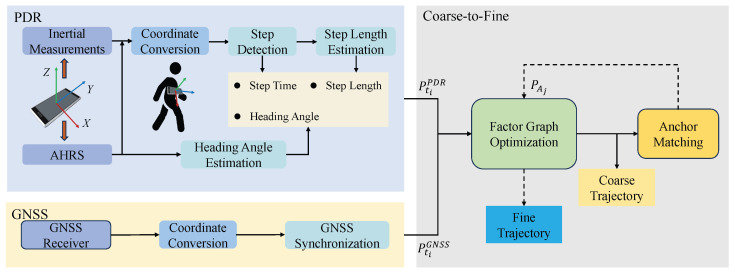
An overview of the proposed FGO-GNSS/PDR localization algorithm. The framework consists of three main components: PDR, GNSS, and a coarse-to-fine optimization strategy. The PDR module estimates pedestrian motion based on inertial and AHRS data, while the GNSS module provides satellite-based absolute positioning. The coarse-to-fine strategy progressively refines the trajectory by integrating multi-source information to optimize localization accuracy.

The overview of the proposed anchor-aided FGO-GNSS/PDR localization algorithm is illustrated in Figure 2. The framework adopts a coarse-to-fine optimization strategy inspired by techniques commonly used in computer vision. Initially, pedestrian motion is estimated using a PDR algorithm driven by inertial and AHRS data from the smartphone. The PDR process includes coordinate transformation from the device frame to the East–North–Up (ENU) frame, step detection, step length estimation, and heading estimation. Next, smartphone GNSS positioning measurements are combined with the PDR results to construct an initial factor graph, producing a coarse localization estimate along the pedestrian trajectory. This preliminary trajectory is then automatically matched to predefined road anchors. The resulting anchor constraints are incorporated into the graph for fine-grained optimization, yielding the final localization result. Notably, all optimization is conducted in the ENU coordinate frame.

### 2.1. PDR Mechanism

**Figure 3:**
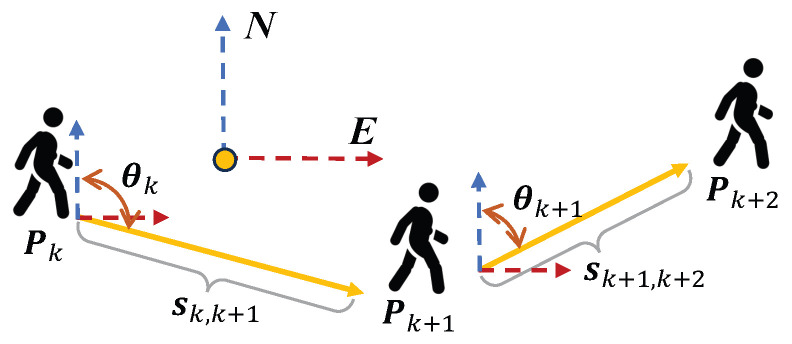
PDR mechanism.

In the PDR process, the algorithm estimates the pedestrian’s relative displacement by detecting step events, estimating the step length and heading direction at each step. By accumulating these step-wise displacements, the complete pedestrian trajectory can be reconstructed. Figure 3 illustrates the mechanism of position updates. In the ENU coordinate frame, this process can be mathematically expressed as follows:

Assuming the initial position is $P_{0}$,

(1) $$P_{0}=\left[\begin{array}{c} E_{0}\\ N_{0} \end{array}\right]$$

The position update at step k+1 can then be expressed as

(2) $$P_{k+1}=\left[\begin{array}{c} E_{k+1}\\ N_{k+1} \end{array}\right]=P_{k}+s_{k,k+1}\cdot \left[\begin{array}{c} \sin(\theta_{k,k+1})\\ \cos(\theta_{k,k+1}) \end{array}\right]$$

where $P_{k}$ denotes the pedestrian position at the beginning of step k+1 and $P_{k+1}$ represents the updated position after the step. $E_{k+1}$ and $N_{k+1}$ correspond to the East and North components in the ENU coordinate frame. $s_{k,k+1}$ is the estimated step length, and $\theta_{k,k+1}$ is the heading angle of the current step with respect to the north direction.

#### 2.1.1. PDR Modeling

**Figure 4:**
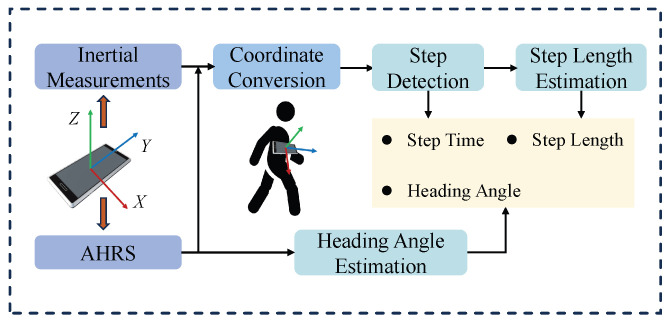
An overview of the proposed PDR algorithm framework, including coordinate conversion, step detection, step length estimation, and heading estimation.

In this study, the smartphone is placed on the user’s chest, with the Y-axis aligned vertically in the walking direction. Compared to the waist, the chest offers a more stable and centrally aligned mounting position, reducing the influence of lateral sway. Although this setup may slightly compromise step-frequency sensitivity, it enhances heading stability and is more practical for real-world deployment in remote assistance scenarios. The overall PDR framework is illustrated in Figure 4. We use the smartphone’s accelerometer and AHRS outputs as inputs.

The accelerometer output *a* is transformed to the ENU coordinate frame using the rotation matrix *R* from the AHRS system. The transformation from the smartphone’s device frame to the ENU frame is defined as follows:

(3) $$R_{\mathrm{body}}^{\mathrm{ENU}}=R_{z}\;@\;R_{x}\;@\;R_{y}$$

Here, $R_{\mathrm{body}}^{\mathrm{ENU}}$ denotes the rotation matrix that transforms vectors from the smartphone coordinate frame to the ENU coordinate frame. $R_{x}$, $R_{y}$, and $R_{z}$ are rotation matrices derived from the Euler angles provided by the AHRS system, and the symbol @ indicates matrix multiplication.

The PDR algorithm consists of three main steps: step detection, step length estimation, and heading estimation, which are described below:

(a) Step Detection: The algorithm detects steps by identifying valleys in the vertical acceleration signal. A fixed threshold is set to filter out noise and avoid false detections.
(b) Step Length Estimation: Step length is estimated using the Weinberg method [44] based on the peak and valley values of the vertical acceleration during a step. The empirical formula is given as
  (4) $$l=K_{w}\cdot \sqrt[{4}]{a_{v,\max}-a_{v,\min}}$$
  where *l* denotes the estimated step length, $K_{w}$ is a step length coefficient, and $a_{v,\max}$ and $a_{v,\min}$ represent the maximum and minimum vertical acceleration values within a step.
(c) Heading Estimation: The heading angle is estimated using the yaw output from the smartphone’s AHRS, corrected by an offset angle of −90° in our placement scenario. To mitigate fluctuations caused by body sway, a moving average filter is applied to smooth the yaw signal. Since direct angle averaging may lead to errors near the $\pm 180^{{}^{\circ}}$ boundary, we project the yaw angles onto the unit circle, average the projected values, and then convert the result back to an angle.

#### 2.1.2. PDR Factor

The PDR model described above produces horizontal displacements in the ENU coordinate frame at each step. Given an initial position, the full pedestrian trajectory can be obtained. In our factor graph optimization framework, the PDR factor imposes a relative motion constraint between two consecutive positions.

The horizontal displacement between two consecutive steps is defined as

(5) $$\Delta P_{t_{i}}=P_{t_{i}}-P_{t_{i-1}}$$

where $P_{t_{i}}$ and $P_{t_{i-1}}$ denote the pedestrian positions at timestamps $t_{i}$ and $t_{i-1}$, respectively, in the ENU coordinate frame. Thus, $\Delta P_{t_{i}}$ represents the relative displacement between two consecutive time steps.

The formulation of the PDR factor is then given by

(6) $${∥e_{t_{i}}^{\mathrm{PDR}}∥}_{\Sigma_{t_{i}}^{\mathrm{PDR}}}^{2}={∥\Delta P_{t_{i}}^{\mathrm{PDR}}-\Delta P_{t_{i}}∥}_{\Sigma_{t_{i}}^{\mathrm{PDR}}}^{2}$$

Here, $\Delta P_{t_{i}}^{\mathrm{PDR}}$ denotes the relative displacement estimated by the PDR algorithm at time $t_{i}$, and $\Delta P_{t_{i}}$ is the actual displacement between two adjacent positions as described above. $e_{t_{i}}^{\mathrm{PDR}}$ is the residual of the PDR factor at timestamp $t_{i}$, and $\Sigma_{t_{i}}^{\mathrm{PDR}}$ is the covariance matrix of the PDR output at time $t_{i}$. In our FGO framework, each PDR factor models the 2D relative displacement on the horizontal plane (E and N). To simplify the modeling, we assume that the noise in the E and N directions is uncorrelated and identically distributed. Accordingly, we adopt an isotropic noise model, represented by a fixed diagonal covariance matrix:

(7) $$\Sigma_{t_{i}}^{PDR}=\sigma_{PDR}^{2}\left[\begin{array}{cc} 1 & 0\\ 0 & 1 \end{array}\right]$$

The value of $\sigma_{PDR}$ is empirically determined based on the observed noise characteristics of the PDR output in our datasets. While more complex noise models could be applied, this simplified isotropic formulation enables efficient optimization while maintaining robust performance.

### 2.2. Anchor-Matching Method

To improve the localization accuracy, the proposed method incorporates anchor constraints derived from the available road geometry, specifically at turning points. These anchors serve as auxiliary constraints to correct the fused trajectory during optimization. Each anchor is placed at the geometric center of a turning region, with its true geographic coordinates (latitude and longitude) obtained through prior measurement. When the pedestrian trajectory passes through such a region, the corresponding anchor is automatically matched based on the local trajectory geometry.

To match each predefined anchor point with the corresponding pedestrian location along the trajectory, the method first selects a set of candidate points from the coarse localization result. Specifically, all trajectory points between the first and last points that lie within a 10 m radius of the anchor are selected, ensuring spatial continuity along the path.

Assume that *N* candidate points are obtained for a given anchor. From these *N* points, the method computes N−1 forward-direction vectors ${\vec{v}}_{i}$ representing the pedestrian’s heading between consecutive points:

(8) $${\vec{v}}_{i}=P_{i+1}-P_{i},\;i=1,2,\ldots ,N-1$$

Then, the angular differences (turning angles) between adjacent heading vectors are calculated to produce N−2 direction change values $\delta \theta_{i}$:

(9) $$\delta \theta_{i}=∠\left({\vec{v}}_{i},{\vec{v}}_{i+1}\right),\;i=1,2,\ldots ,N-2$$

A fixed-length sliding window of size *W* is applied to the turning angle sequence. For each window, the cumulative turning angle $\Delta \theta_{j}$ is computed as

(10) $$\Delta \theta_{j}=\sum_{i=j}^{j+W-3}\delta \theta_{i},\;j=1,2,\ldots ,N-W+1$$

**Figure 5:**
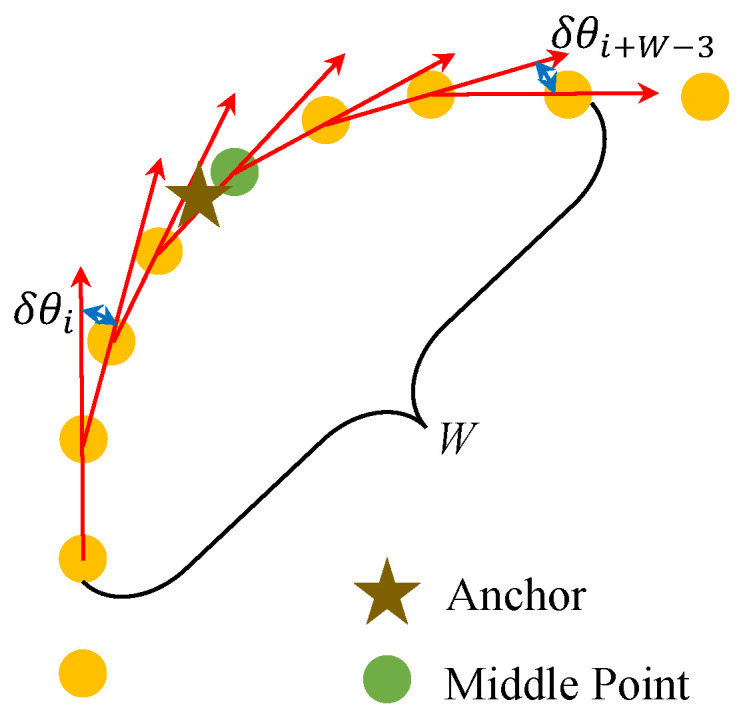
Anchor-matching process: Turning angle accumulation is computed within a fixed-length sliding window. The middle point of the window with the largest cumulative angle change is selected as the anchor match.

As illustrated in Figure 5, the window with the maximum cumulative turning angle is identified, and its center point is selected as the matched location for the current anchor. Formally, the index of the matched point $n_{anchor}$ is determined as

(11) $$n_{\mathrm{anchor}}=j^{*}+\left\lfloor \frac{W}{2}\right\rfloor ,\;\mathrm{where}\;j^{*}=\arg\max_{j}\Delta \theta_{j}$$

This method ensures that anchor points are matched to the portions of the trajectory exhibiting the most significant local turning behavior, improving the robustness and accuracy of anchor alignment in complex environments.

### 2.3. Anchor Factor

To further improve localization accuracy, we introduce an anchor factor that leverages known geometric landmarks, specifically road turning points, as additional spatial constraints in the factor graph. These anchors serve as reference points to mitigate accumulated drift from GNSS and PDR estimates.

The anchor factor is defined as a residual between the estimated pedestrian position and the known ENU position of the corresponding anchor point:

(12) $${∥e_{t_{a_{k}}}^{\mathrm{Anchor}}∥}_{\Sigma_{t_{a_{k}}}^{\mathrm{Anchor}}}^{2}={∥P_{a_{k}}^{\mathrm{Anchor}}-P_{t_{a_{k}}}∥}_{\Sigma_{t_{a_{k}}}^{\mathrm{Anchor}}}^{2}$$

Here, $P_{a_{k}}^{\mathrm{Anchor}}$ denotes the true ENU coordinate of the anchor $a_{k}$, and $P_{t_{a_{k}}}$ is the estimated pedestrian position at time $t_{a_{k}}$ when matched to this anchor. The residual $e_{t_{a_{k}}}^{\mathrm{Anchor}}$ quantifies the positional deviation between the estimated and ground-truth anchor location. The covariance matrix $\Sigma_{t_{a_{k}}}^{\mathrm{Anchor}}$ models the uncertainty associated with anchor matching. Similar to the PDR factor, the anchor factor also adopts an isotropic noise model. Specifically, for a matched anchor $a_{k}$, the anchor factor models the 2D positional deviation between the estimated pedestrian position $P_{t_{a_{k}}}$ and the ground-truth anchor position $P_{a_{k}}^{\mathrm{Anchor}}$ on the horizontal plane (E and N). We assume that the uncertainties in the E and N directions are uncorrelated and identically distributed, allowing the covariance matrix $\Sigma_{t_{a_{k}}}^{\mathrm{Anchor}}$ to be represented as a fixed diagonal matrix: 

(13) $$\Sigma_{t_{a_{k}}}^{\mathrm{Anchor}}=\sigma_{\mathrm{Anchor}}^{2}\left[\begin{array}{cc} 1 & 0\\ 0 & 1 \end{array}\right]$$

The parameter $\sigma_{\mathrm{Anchor}}$ is empirically determined based on the variability observed in anchor-matching performance in our datasets.

This anchor factor enhances the robustness of trajectory optimization by introducing strong spatial constraints in turning regions, where PDR performance tends to deteriorate. In our current implementation, the start and end positions are assumed to be approximately known, based on external information such as commercial maps or fixed landmarks (e.g., building entrances). This simplifying assumption reflects common RSA scenarios, where the origin and destination are often predefined. While this improves the stability of FGO by providing fixed anchor constraints, we acknowledge that it may not hold in all real-world cases. In future work, we aim to relax this assumption by integrating visual place recognition techniques, such as OrienterNet [45], to support autonomous initialization and termination without relying on predefined points. These points are also treated as anchor nodes and incorporated into the factor graph as prior constraints. The formulation of their residuals follows the same structure as that of regular anchors.

### 2.4. GNSS Factor

GNSS positioning provides absolute spatial references, serving as global constraints in the optimization process. To incorporate this information, we introduce GNSS factors into the factor graph. In our implementation, the GNSS positions are obtained from the Android device solution [46]. First, the device solution results are transformed from geodetic coordinates (latitude, longitude, height) to the Earth-Centered Earth-Fixed (ECEF) coordinate system. To simplify the transformation, a fixed height is assumed in this work, which is reasonable in flat urban areas but may introduce errors in hilly terrains. Then, given a known reference point, the rotation matrix $R_{\mathrm{ECEF}}^{\mathrm{ENU}}$ is computed to convert the ECEF coordinates into the local ENU frame. The GNSS factor is formulated as

(14) $${∥e_{t_{i}}^{\mathrm{GNSS}}∥}_{\Sigma_{t_{i}}^{\mathrm{GNSS}}}^{2}={∥P_{t_{i}}^{\mathrm{GNSS}}-P_{t_{i}}∥}_{\Sigma_{t_{i}}^{\mathrm{GNSS}}}^{2}$$

Here, $P_{t_{i}}^{\mathrm{GNSS}}$ represents the GNSS position at time $t_{i}$, and $P_{t_{i}}$ denotes the estimated pedestrian position at the same timestamp. The residual $e_{t_{i}}^{\mathrm{GNSS}}$ measures the deviation between the GNSS position and the current state estimate. Similar to the PDR and anchor factors, the GNSS factor also adopts an isotropic noise model. Specifically, for a GNSS observation at time $t_{i}$, the GNSS factor models the 2D positional deviation between the estimated pedestrian position $P_{t_{i}}$ and the GNSS position $P_{t_{i}}^{\mathrm{GNSS}}$ on the horizontal plane (E and N). We assume that the errors in the E and N directions are uncorrelated and identically distributed, so the covariance matrix $\Sigma_{t_{i}}^{\mathrm{GNSS}}$ can be represented as a fixed diagonal matrix:

(15) $$\Sigma_{t_{i}}^{\mathrm{GNSS}}=\sigma_{\mathrm{GNSS}}^{2}\left[\begin{array}{cc} 1 & 0\\ 0 & 1 \end{array}\right]$$

The value of $\sigma_{\mathrm{GNSS}}$ is empirically determined from the observed noise characteristics of GNSS positioning in our datasets.

### 2.5. Factor Graph Integration

In the proposed optimization framework, we adopt a coarse-to-fine strategy. Initially, the GNSS factor, PDR factor, and start&end anchor constraints are used to perform a coarse optimization and obtain an initial trajectory estimate. The corresponding loss function is defined as

(16) $$\hat{P}=\arg\min_{P}\sum_{t_{i}}\left(e_{t_{i}}^{\mathrm{PDR}}+e_{t_{i}}^{\mathrm{GNSS}}+e_{start\&end}^{\mathrm{Anchor}}\right)$$

The resulting trajectory is then used as input to the anchor-matching algorithm, which determines matched anchor positions. These anchor constraints are subsequently incorporated into the factor graph for fine-grained optimization. The final objective function for the complete factor graph optimization is given by

(17) $$\hat{P}=\arg\min_{P}\sum_{t_{i},t_{a_{k}}}\left(e_{t_{i}}^{\mathrm{PDR}}+e_{t_{a_{k}}}^{\mathrm{Anchor}}+e_{t_{i}}^{\mathrm{GNSS}}\right)$$

**Figure 6:**
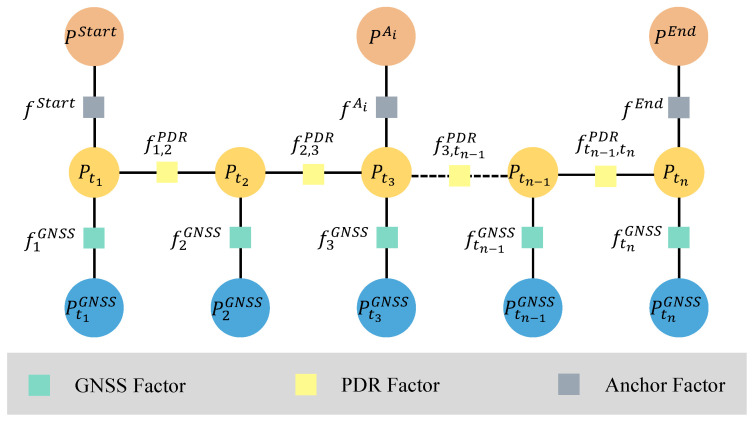
The structure of the final factor graph, including GNSS factors (green), PDR factors (light yellow), and anchor factors (gray).

The optimized trajectory $\hat{P}$ represents the best estimate of the pedestrian position at each timestamp $t_{i}$ based on the fused constraints. The structure of the final factor graph is illustrated in Figure 6. It consists of three types of factors: PDR, anchor, and GNSS. In our implementation, we use the Georgia Tech Smoothing and Mapping (GTSAM) library [47], an open-source optimization framework developed by Georgia Institute of Technology, to perform factor graph optimization. The Levenberg–Marquardt algorithm [48] is applied to solve the entire nonlinear optimization problem.

## 3. Results

### 3.1. Experiment Setup

To validate the effectiveness of the proposed method, we collected real-world data in three challenging urban environments. These environments feature dense buildings and vegetation, which induce GNSS positioning errors due to multipath propagation and NLOS conditions. These characteristics pose significant challenges for GNSS localization.

In addition, we intentionally used a consumer-grade smartphone (OPPO PESM10, manufactured by OPPO Guangdong Mobile Telecommunications Corp., Ltd., Dongguan, China) rather than the high-end devices commonly adopted in similar studies [14,16,17]. The device supports multi-constellation GNSS, including GPS, GLONASS, BeiDou, Galileo, QZSS, and A-GPS. GNSS data were recorded at a frequency of 1 Hz using the Android fused location provider. The smartphone is also equipped with a typical suite of inertial sensors: a 3-axis accelerometer, a 3-axis gyroscope, and a 3-axis magnetometer. The smartphone data were collected using the GetSensorData app [46], which records inertial and AHRS outputs at 50 Hz. The GNSS data were logged at a frequency of 1 Hz.

**Figure 7:**
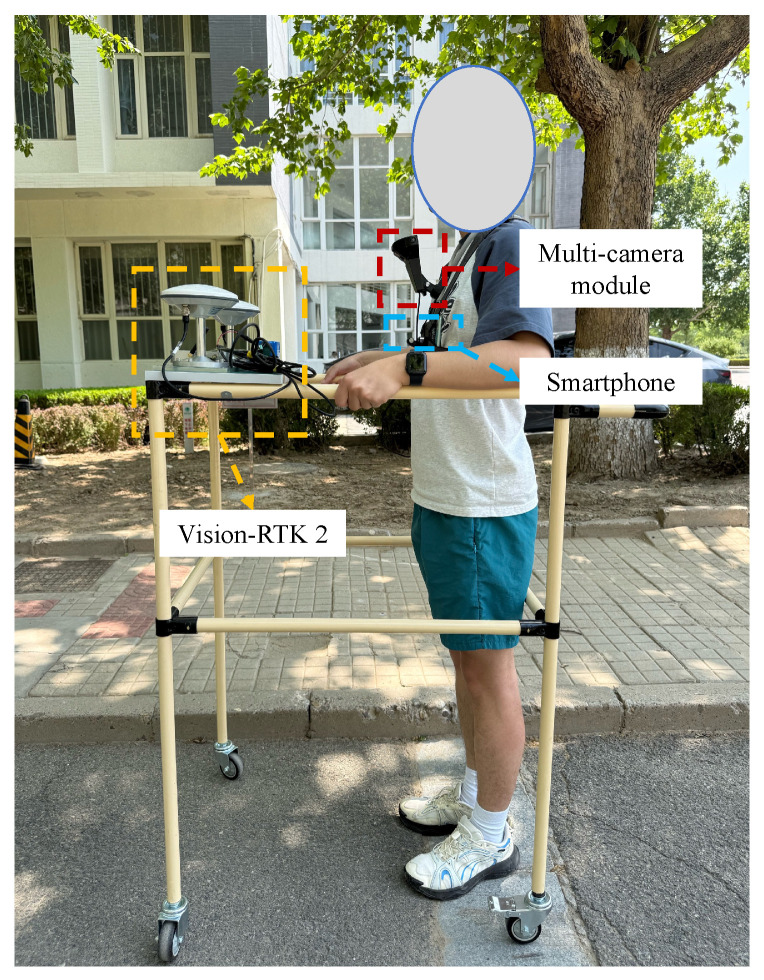
Data acquisition setup. The system consists of a smartphone and a multi-camera module for data collection and a Vision-RTK 2 unit for recording ground-truth trajectories.

The data acquisition setup is depicted in Figure 7. The smartphone was chest-mounted on the user, following the configuration described in the methodology section. For ground-truth acquisition, we employed a Vision-RTK 2 system that integrates visual-inertial odometry with RTK corrections to achieve centimeter-level accuracy. As shown in Figure 7, the RTK receiver was mounted on a wheeled platform moving alongside the user, enabling accurate reference trajectory collection at 10 Hz. All experiments were performed on a desktop computer equipped with an Intel i5-13490F 2.5 GHz CPU and 32 GB of RAM, running Ubuntu 24.04.

### 3.2. Evaluation Metrics and Methods

We adopt four commonly used evaluation metrics to evaluate the performance of the proposed method: mean error (MEAN), standard deviation (STD), root mean square error (RMSE), and maximum error (MAX). All evaluations are performed in the ENU coordinate frame. To validate the effectiveness of the proposed anchor factor, we compare the localization results of the following three methods:

1. GNSS: The GNSS positions are obtained from the on-chip fused GNSS location estimates provided by the Android device via the GetSensorData app.
2. FGO-GNSS/PDR: Localization results obtained from the coarse optimization stage using GNSS, PDR, and start&end anchor constraints.
3. FGO-GNSS/PDR + Anchor: The final localization results obtained by incorporating the proposed anchor factor into the factor graph and applying the complete coarse-to-fine optimization strategy.

We acknowledge that all experiments in this study were conducted using a single smartphone model (OPPO PESM10), which may limit the generalizability of the results across different devices. Variations in GNSS hardware, antenna quality, and sensor fusion implementations among smartphone models could influence positioning performance. In future work, we plan to investigate the robustness and adaptability of the proposed method across multiple device types to assess cross-device generalization.

### 3.3. Experiment 1 in Urban Areas

**Figure 8:**
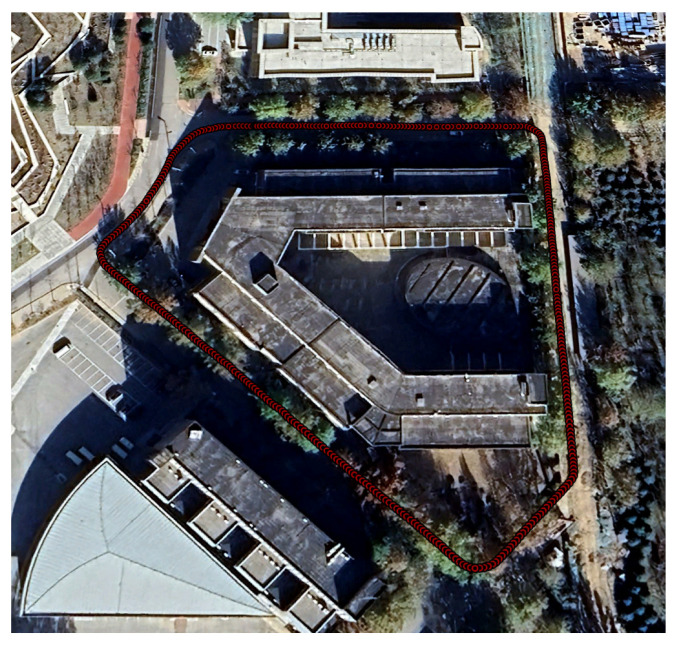
A scene map and trajectory layout for Experiment 1, visualized in Google Earth [49]. The trajectory shown represents the ground truth, collected using the Vision-RTK 2, with red circles denoting pedestrian positions sampled at one-second intervals.

To evaluate the effectiveness of the proposed method, we selected a representative and challenging urban environment. The scene map and corresponding trajectory layout is shown in Figure 8. The path includes five turning anchors, where PDR performance typically deteriorates due to heading estimation errors and changes in gait dynamics introduced by turning motions. This makes the environment particularly suitable for evaluating the effectiveness of the proposed anchor factor. In this area, we collected three walking datasets with durations of 376 s, 396 s, and 397 s and corresponding step counts of 608, 616, and 629, respectively.

**Figure 9:**
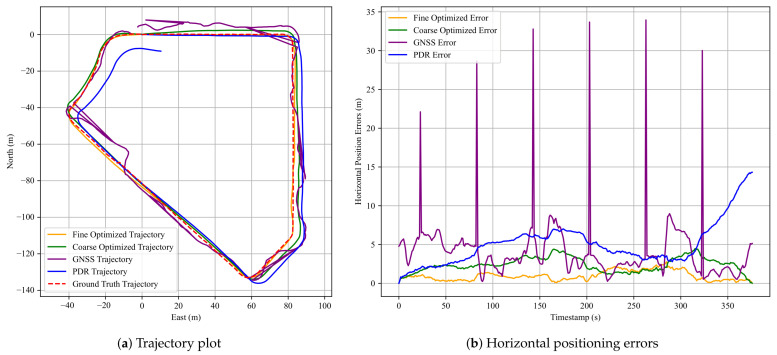
Results for the first dataset in Experiment 1. (**a**) A comparison of pedestrian trajectories obtained using the three methods; (**b**) corresponding horizontal positioning errors over time. The red dashed line denotes the ground truth, the blue solid line indicates the PDR result, the purple solid line represents the GNSS result, the green solid line shows the FGO-GNSS/PDR result, and the orange solid line corresponds to the FGO-GNSS/PDR + Anchor result.

Figure 9 presents the corresponding pedestrian trajectories and the horizontal positioning errors for the first dataset. In the trajectory plot, the red dashed line denotes the ground-truth trajectory, the purple solid line shows the raw GNSS trajectory, the green solid line illustrates the result of FGO-GNSS/PDR, and the orange solid line depicts the final output after incorporating the proposed anchor factor. As observed, the FGO-GNSS/ PDR + Anchor method yields a trajectory that closely aligns with the ground-truth trajectory, while both the raw GNSS and FGO-GNSS/PDR trajectories exhibit noticeable deviations. In particular, the GNSS result shows substantial drift. This observation is further supported by the horizontal error plot, where the raw GNSS solution reaches a peak error of 33.95 m. Additional results are visualized in Appendix A.1.

Table 1 summarizes the localization errors of the three methods using four standard metrics: RMSE, MEAN, STD, and MAX. Table 2 presents the performance improvement of the FGO-GNSS/PDR + Anchor method compared to the baseline FGO-GNSS/PDR approach. The performance gap between raw GNSS and the optimized methods highlights the limitations of GNSS localization in urban environments. The raw GNSS trajectories suffer from large deviations, particularly in areas surrounded by tall buildings or vegetation, with maximum errors exceeding 30 m. Although the FGO-GNSS/PDR method effectively suppresses noise by leveraging inertial data, it still experiences drift over time due to the absence of reliable global constraints, as is evident in the third dataset, where the maximum error remains above 11 m.

In contrast, the proposed FGO-GNSS/PDR + Anchor method consistently achieves lower localization errors across all datasets. The maximum errors are reduced to below 3.1 m, and the error distributions are notably tighter. The significantly lower standard deviations indicate not only improved accuracy but also greater stability and robustness. This demonstrates the value of incorporating anchor constraints derived from known road geometry, especially in turning regions where PDR is prone to degradation.

The third dataset exhibits the most substantial improvement, with RMSE reduced by 71%, MEAN by 65%, STD by 82%, and MAX error by 76% compared to the FGO-GNSS/PDR baseline. These results suggest that the proposed anchor factor is particularly beneficial in complex urban scenarios involving multiple turns, where it can provide tighter constraints on trajectory estimation.

Moreover, the consistent improvements observed across all three datasets demonstrate the potential of the proposed method in structured urban environments. The error reduction rates suggest that anchor-aided optimization not only decreases average errors but also effectively suppresses large deviations. Nevertheless, further evaluation across more diverse routes and scenarios is needed to validate its generalization and robustness.

### 3.4. Experiment 2 in Urban Areas

**Figure 10:**
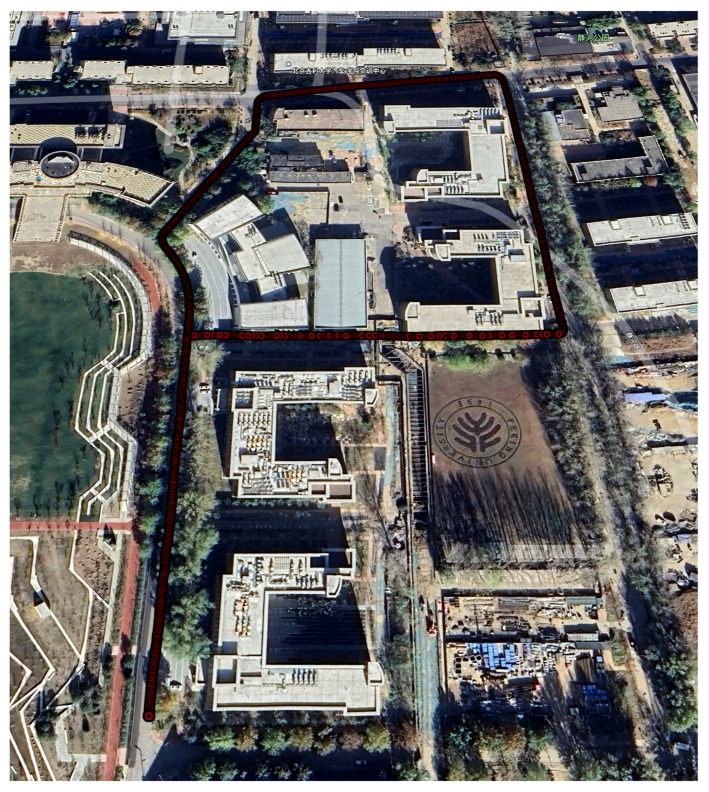
Scene map and trajectory layout for Experiment 2, visualized in Google Earth. The trajectory shown represents the ground truth, collected using the Vision-RTK 2, with red circles denoting pedestrian positions sampled at one-second intervals.

In Experiment 2, we selected a more challenging long-range test environment featuring curves and an extended walking path. The scene map and corresponding trajectory layout are shown in Figure 10. This setting allows for a more comprehensive evaluation of the robustness of the proposed anchor-aided localization approach. Three datasets were collected with walking durations of 871 s, 904 s, and 890 s and corresponding step counts of 1274, 1324, and 1317, respectively.

**Figure 11:**
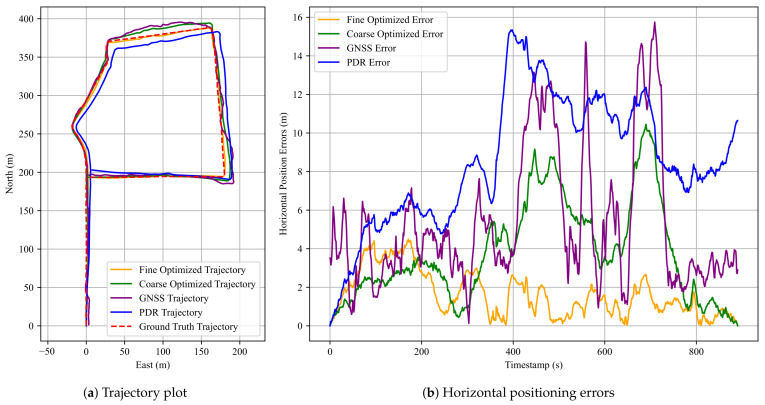
Results for the third dataset in Experiment 2. (**a**) A comparison of pedestrian trajectories obtained using the three methods; (**b**) corresponding horizontal positioning errors over time. The red dashed line denotes the ground truth, the blue solid line indicates the PDR result, the purple solid line represents the GNSS result, the green solid line shows the FGO-GNSS/PDR result, and the orange solid line corresponds to the FGO-GNSS/PDR + Anchor result.

Similar to Experiment 1, Figure 11 presents the pedestrian trajectories and horizontal positioning errors for the third dataset. Table 3 summarizes the positioning performance (RMSE, MEAN, STD, MAX) of all three methods, while Table 4 shows the improvement rates of the FGO-GNSS/PDR + Anchor method compared to the baseline FGO-GNSS/PDR. Consistent with previous results, the proposed FGO-GNSS/PDR + Anchor method achieves the highest accuracy across all datasets. Additional results are provided in Appendix A.2.

As shown in the horizontal error plot, the FGO-GNSS/PDR method still exhibits considerable deviations from the ground-truth trajectory, particularly in the third dataset, where the maximum error reaches 10.44 m. In contrast, the FGO-GNSS/PDR + Anchor method effectively reduces the maximum error to 4.49 m—a reduction of 57%. Likewise, the mean error decreases from 3.79 m to 1.63 m, representing a 57% improvement. These results confirm the effectiveness of anchor constraints in correcting accumulated drift in complex scenes. In the first dataset, the best mean error achieved is only 1.54 m, and the STD is as low as 0.93 m. These results further confirm the effectiveness of the proposed anchor factor. These findings demonstrate that the proposed anchor factor significantly improves both the accuracy and stability of position estimation.

An interesting observation can be made in Figure 11b, where the fine-optimized trajectory exhibits a slightly higher positioning error than the coarse-optimized trajectory during the 0–200 s interval of the third dataset. This behavior may be attributed to the redistribution of constraint weights in the factor graph. Specifically, during fine optimization, the relative influence of GNSS constraints decreases due to the incorporation of additional anchor constraints. While this adjustment improves robustness in challenging areas, it reduces the contribution of reliable GNSS data in well-performing segments. Since the GNSS signal quality is relatively high in the initial 0–200 s, the reduced contribution of GNSS data may lead to a marginal degradation in accuracy. This trade-off highlights the importance of appropriately balancing the contributions of anchor and GNSS factors.

Overall, the results of Experiment 2 demonstrate the effectiveness of our approach under similar walking conditions. While the method shows robustness to small path deviations and heading variations, further evaluation on more diverse walking patterns is necessary to fully validate its generalizability. It also shows that the proposed anchor-aided FGO-GNSS/PDR remains effective in extended-range, geometrically complex urban settings, thereby highlighting the method’s suitability for real-world pedestrian localization applications.

### 3.5. Experiment 3 in Urban Areas

**Figure 12:**
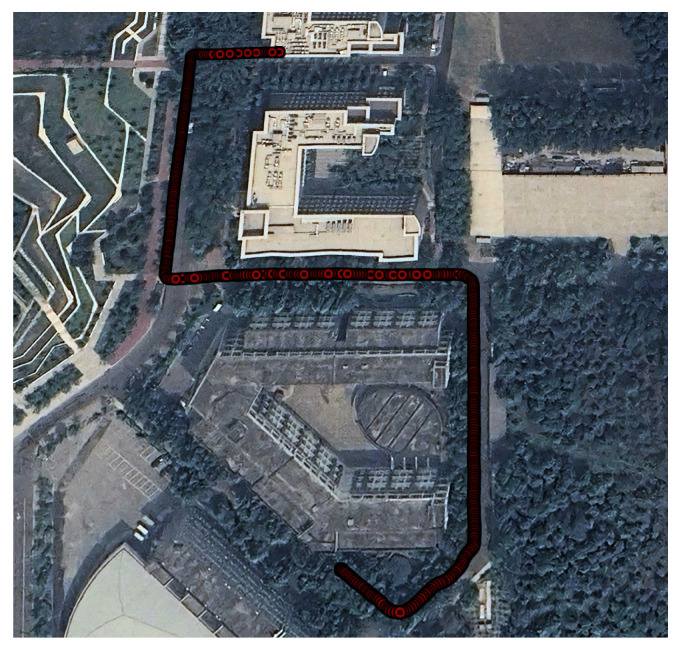
Scene map and trajectory layout for Experiment 3, visualized in Google Earth. The trajectory shown represents the ground truth, collected using the Vision-RTK 2, with red circles denoting pedestrian positions sampled at one-second intervals.

In Experiment 3, we collected a dataset that contains a partial trajectory overlapping with that in Experiment 1. The corresponding scene map and trajectory layout are illustrated in Figure 12. This trajectory includes three anchor points from Experiment 1 and two additional newly deployed anchors, enabling a denser spatial constraint distribution. The dataset was recorded with a total walking duration of 434 s, covering a step count of 658.

**Figure 13:**
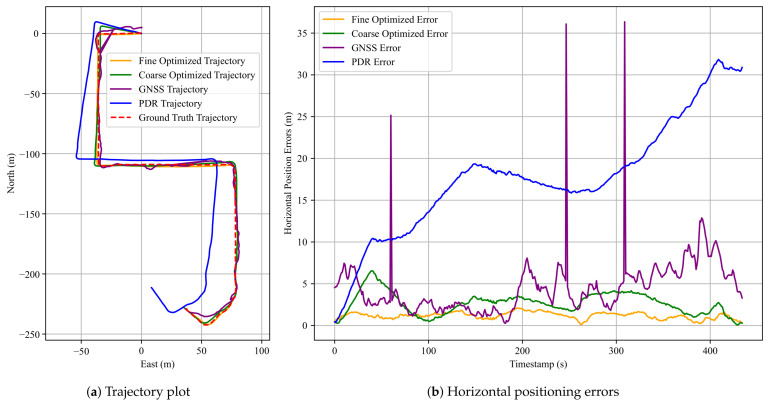
Results for the dataset in Experiment 3. (**a**) A comparison of pedestrian trajectories obtained using the three methods; (**b**) corresponding horizontal positioning errors over time. The red dashed line denotes the ground truth, the blue solid line indicates the PDR result, the purple solid line represents the GNSS result, the green solid line shows the FGO-GNSS/PDR result, and the orange solid line corresponds to the FGO-GNSS/PDR + Anchor result.

The pedestrian trajectories and horizontal positioning errors for this dataset are presented in Figure 13, while the quantitative positioning performance is summarized in Table 5 and Table 6. As depicted in Figure 13a, the PDR trajectory exhibits large angular deviations similar to those observed in dataset 3 of Experiment 1, indicating accumulated heading drift. Nevertheless, after fine optimization, the resulting trajectory closely matches the ground truth, highlighting the corrective effect of the anchor constraints. Figure 13b further shows that the fine optimized solution maintains low error levels throughout most of the trajectory, whereas the GNSS and standalone PDR results suffer from significant fluctuations and drifts.

The RMSE, MEAN, STD, and MAX errors are compared across the three evaluated methods. It can be clearly observed that the proposed FGO-GNSS/PDR + Anchor method yields the most accurate results, with trajectories that are well-aligned with the ground-truth path. Specifically, as shown in Table 5, the maximum horizontal error achieved by the proposed method is only 2.10 m, while the mean horizontal error is reduced to 1.19 m. Compared with the GNSS-only and FGO-GNSS/PDR methods, the proposed anchor-assisted approach consistently achieves substantial reductions in all error metrics. The RMSE, MEAN, STD, and MAX values are reduced by 58%, 55%, 70%, and 68%, respectively, relative to the FGO-GNSS/PDR baseline. These results verify that incorporating anchor factors not only improves overall accuracy but also enhances the stability of trajectory estimation in challenging urban environments.

## 4. Discussion

### 4.1. PDR Results

**Figure 14:**
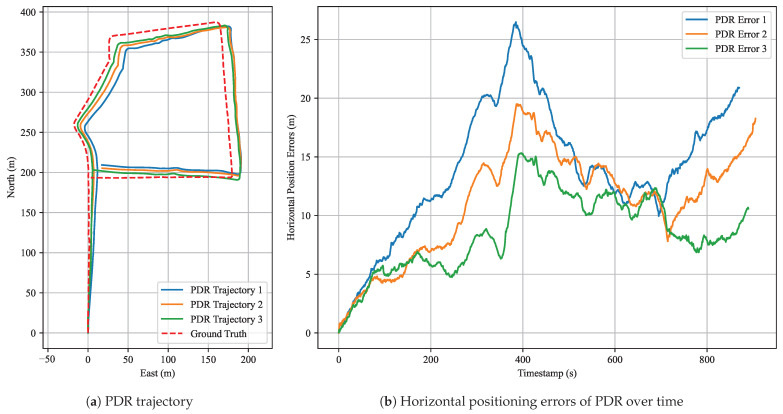
PDR performance in Experiment 2. (**a**) Reconstructed trajectory using the proposed PDR algorithm; (**b**) corresponding horizontal positioning errors over time for three datasets.

Figure 14 shows the trajectory and horizontal error distribution of the PDR algorithm in Experiment 2. This figure is intended to illustrate the standalone positioning performance of the PDR algorithm. Blue, orange, and green curves correspond to the results of the first, second, and third datasets, respectively. From the trajectory plots, we observe that the estimated PDR trajectories generally align well with the ground-truth path in shape, although deviations of varying magnitudes exist. Notably, near the turning points, the proposed PDR algorithm accurately and smoothly reconstructs the original curves, providing reliable inputs for anchor matching.

Statistical results (Table 7) for the three datasets show that the third dataset yields the best performance, with a mean error of 8.53 m and RMSE of 9.17 m. The first dataset exhibits the largest deviation, with a mean error of 13.93 m and RMSE of 15.07 m. These results demonstrate that the proposed PDR algorithm provides accurate local relative motion estimates, particularly suitable for reconstructing the shape of walking trajectories.

Nevertheless, as PDR errors accumulate over time, it cannot ensure long-term global accuracy. To mitigate this issue, absolute position constraints from GNSS can be integrated to correct large-scale drifts. The proposed anchor factor enables more precise localization corrections by leveraging known anchor points. Therefore, this paper introduces a coarse-to-fine optimization strategy: we first use GNSS data to roughly align the trajectory to a global reference, and then we apply anchor matching based on the adjusted trajectory to obtain accurate localization constraints.

Improving the quality of PDR results hinges on the ability to obtain accurate heading estimates. Smartphones equipped with high-grade inertial sensors are expected to enable more precise heading estimates and yield lower-error PDR trajectories. Besides improving hardware, developing robust heading estimation algorithms under varying walking postures is equally important.

Future research should explore methods to reduce heading estimation errors caused by body movement and instability. In addition, reliable step detection remains a critical component of PDR systems, and developing approaches to automatically adjust step detection thresholds is an important open question. With the advancement of deep learning techniques, future work can also investigate how learning-based methods may improve step detection, heading estimation, and trajectory reconstruction. Ultimately, the integration of model-based and learning-based approaches represents a viable strategy for enhancing the robustness and accuracy of PDR solutions.

### 4.2. Anchor Effect

To achieve more accurate pedestrian localization, we proposed the anchor factor, which leverages prior knowledge of road geometry to impose constraints during optimization. We also proposed an anchor-matching algorithm that automatically aligns trajectory points with predefined anchors. Taking Experiment 1 as an example, Table 8 summarizes the anchor-matching errors across the three datasets using the anchor-matching method proposed in this study.

Statistical results (Table 9) show that the average anchor-matching error across all datasets remains below 1.1 m. Particularly in the first dataset, the matching RMSE is only 0.21 m and MEAN is 0.19 m. These results demonstrate the effectiveness of the proposed matching algorithm in achieving accurate anchor correspondence.

**Figure 15:**
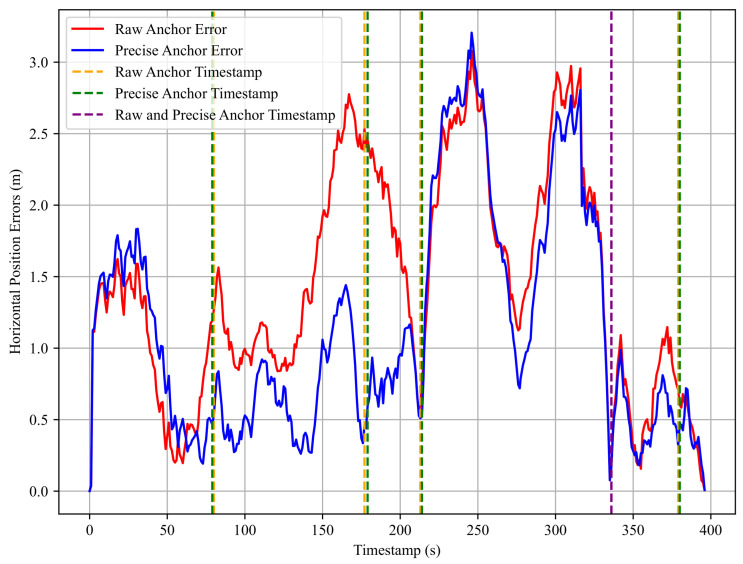
Horizontal positioning errors before and after anchor refinement for the 2nd dataset in Experiment 1. The red line represents errors using the raw anchor positions, while the blue line shows errors after refinement. Orange, green, and purple dashed lines indicate the timestamps of raw anchors, refined anchors, and their shared anchors, respectively.

To further evaluate the effectiveness of the anchor factor, we refined the anchor-matching results for the second dataset in Experiment 1, which exhibited the largest anchor-matching errors. Figure 15 illustrates the horizontal positioning errors before and after anchor refinement. The red line represents the errors using raw anchors obtained directly from the angle-based matching algorithm, while the blue line shows the errors after refinement with more accurate anchor positions selected from video frames. Orange dashed lines mark the timestamps of raw anchors, green dashed lines indicate the timestamps of refined anchors, and purple dashed lines represent timestamps shared by both raw and refined anchors.

As shown in the error plot, the horizontal positioning errors significantly decrease after refining the anchors with previously large matching errors. In particular, between Anchor_1_ and Anchor_2_, the horizontal errors are noticeably reduced following the refinement. According to Table 10, the mean error decreased from 1.45 m to 1.14 m, representing a 21% improvement, while the RMSE dropped from 1.65 m to 1.38 m, corresponding to a 16% reduction. Although the maximum error increased slightly by 0.13 m, this change is negligible and does not affect the overall improvement. These results confirm that accurate anchor matching contributes to better trajectory optimization, significantly reducing localization error. They also highlight the effectiveness of the proposed anchor factor in improving overall localization accuracy.

Nevertheless, the anchor-matching algorithm still has certain limitations. In some scenarios, relatively large matching errors may persist. Experimental results confirm that more accurate anchor positions lead to better localization performance. Therefore, future work may focus on improving the anchor-matching algorithm and exploring strategies for selecting or refining anchor positions to achieve optimal performance.

### 4.3. Another-User Test

**Figure 16:**
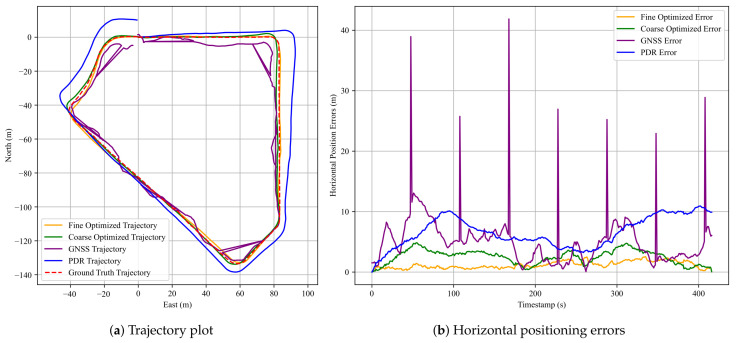
Results for the another-user dataset. (**a**) A comparison of pedestrian trajectories obtained using the three methods; (**b**) corresponding horizontal positioning errors over time. The red dashed line denotes the ground truth, the blue solid line indicates the PDR result, the purple solid line represents the GNSS result, the green solid line shows the FGO-GNSS/PDR result, and the orange solid line corresponds to the FGO-GNSS/PDR + Anchor result.

We further evaluated the robustness of our method by collecting an additional dataset from another user in the same test environment as Experiment 1. Figure 16 shows the trajectory and horizontal error comparisons among different methods.

As shown in the trajectory plot (Figure 16a), the FGO-GNSS/PDR + Anchor method yields the trajectory that closely follows the ground-truth path, consistent with the performance observed in Experiments 1 and 2. In the corresponding horizontal positioning errors plot (Figure 16b), the error distribution of the FGO-GNSS/PDR + Anchor method is significantly more stable than that of the GNSS and FGO-GNSS/PDR methods. The STD is only 0.58 m, representing a 50% reduction compared to the FGO-GNSS/PDR method. Notably, the original GNSS trajectory exhibits seven major error spikes, all of which are effectively mitigated by the proposed method.

Table 11 summarizes the RMSE, MEAN, STD, and MAX errors for this dataset. Additionally, Table 12 presents the corresponding performance improvements of the FGO-GNSS/PDR + Anchor method relative to the FGO-GNSS/PDR baseline. The dataset exhibits substantial improvement, with RMSE reduced by 52%, MEAN by 53%, STD by 50%, and MAX error by 47% compared to the FGO-GNSS/PDR baseline. These results highlight the accuracy of the proposed method, even when applied to data collected from another user.

## 5. Conclusions

In this paper, we introduce the concept of a video-based map in the context of RSA and propose a pedestrian localization method based on FGO-GNSS/PDR integrated with anchor constraints. To enable automatic anchor matching, we design a coarse-to-fine optimization strategy: GNSS positions, PDR results, and start&end anchors are first fused using factor graph optimization to yield coarse localization estimates. Then, trajectory turning angle cues are leveraged to match anchor points, and anchor-based constraints are incorporated to obtain refined localization results through second-stage optimization. We collected multiple real-world datasets in three representative urban scenarios and conducted both single-user repeated experiments and another-user trials. The experimental results demonstrate that the proposed anchor factor significantly improves localization accuracy across diverse scenarios. Specifically, the introduction of anchor constraints reduced the mean horizontal positioning error by 42% to 65% and the maximum error by 38% to 76% across all datasets. In this study, the mean horizontal positioning error was 1.36 m. Moreover, in the another-user experiment, the method maintained strong robustness, achieving a 47% to 53% reduction in RMSE, MEAN, STD, and MAX errors. Furthermore, the experiments demonstrate the effectiveness of the proposed PDR method in providing accurate local motion constraints. For anchor matching, the trajectory-turning-angle-based approach shows promising performance in structured urban environments, enabling reasonable anchor correspondence under typical conditions. While the method supports video-based map construction and enhances remote assistance capabilities for visually impaired users, its generalizability and robustness across more diverse users and walking patterns require further validation in future studies.

In future work, we plan to incorporate GNSS raw measurements into our framework to achieve higher positioning accuracy. We will enhance the PDR algorithm through improved step detection and heading estimation, enabling more reliable displacement constraints in the FGO framework. In addition, the anchor positions and types will be more precisely defined, and a more robust anchor-matching algorithm will be developed. Finally, we aim to implement a real-time, sliding window-based FGO framework for GNSS/PDR integration to enable real-time pedestrian localization in RSA applications.

## Figures and Tables

**Table 1:** Positioning performance of the listed methods in Experiment 1.

Group	Method	RMSE (m)	MEAN (m)	STD (m)	MAX (m)
1st	GNSS	5.69	4.12	3.93	33.95
	FGO-GNSS/PDR	2.57	2.39	0.95	4.47
	FGO-GNSS/PDR + Anchor	1.15	0.97	0.62	2.77
2nd	GNSS	5.10	4.74	1.88	11.64
	FGO-GNSS/PDR	4.00	3.68	1.58	6.52
	FGO-GNSS/PDR + Anchor	1.65	1.45	0.78	3.08
3rd	GNSS	6.84	5.26	4.37	16.56
	FGO-GNSS/PDR	4.93	3.76	3.19	11.04
	FGO-GNSS/PDR + Anchor	1.43	1.31	0.57	2.61

**Table 2:** Improvement rates of GNSS and FGO-GNSS/PDR + Anchor compared to the baseline in Experiment 1.

Group	Method	RMSE (%)	MEAN (%)	STD (%)	MAX (%)
1st	GNSS	−121%	−72%	−314%	−659%
	FGO-GNSS/PDR	—	—	—	—
	FGO-GNSS/PDR + Anchor	55%	59%	35%	38%
2nd	GNSS	−27%	−29%	−19%	−79%
	FGO-GNSS/PDR	—	—	—	—
	FGO-GNSS/PDR + Anchor	59%	61%	50%	53%
3rd	GNSS	−39%	−40%	−37%	−50%
	FGO-GNSS/PDR	—	—	—	—
	FGO-GNSS/PDR + Anchor	71%	65%	82%	76%

**Table 3:** Positioning performance of the listed methods in Experiment 2.

Group	Method	RMSE (m)	MEAN (m)	STD (m)	MAX (m)
1st	GNSS	6.47	5.59	3.26	24.56
	FGO-GNSS/PDR	4.02	3.51	1.97	7.89
	FGO-GNSS/PDR + Anchor	1.80	1.54	0.93	4.00
2nd	GNSS	7.38	4.90	5.52	39.81
	FGO-GNSS/PDR	3.71	2.83	2.39	9.57
	FGO-GNSS/PDR + Anchor	1.85	1.63	0.87	4.08
3rd	GNSS	6.42	5.35	3.53	15.75
	FGO-GNSS/PDR	4.58	3.79	2.57	10.44
	FGO-GNSS/PDR + Anchor	2.00	1.63	1.15	4.49

**Table 4:** Improvement rates of GNSS and FGO-GNSS/PDR + Anchor compared to the baseline in Experiment 2.

Group	Method	RMSE (%)	MEAN (%)	STD (%)	MAX (%)
1st	GNSS	−61%	−59%	−65%	−211%
	FGO-GNSS/PDR	—	—	—	—
	FGO-GNSS/PDR + Anchor	55%	56%	53%	49%
2nd	GNSS	−99%	−73%	−131%	−316%
	FGO-GNSS/PDR	—	—	—	—
	FGO-GNSS/PDR + Anchor	50%	42%	64%	57%
3rd	GNSS	−40%	−41%	−37%	−51%
	FGO-GNSS/PDR	—	—	—	—
	FGO-GNSS/PDR + Anchor	56%	57%	55%	57%

**Table 5:** Positioning performance of the listed methods in Experiment 3.

Method	RMSE (m)	MEAN (m)	STD (m)	MAX (m)
GNSS	5.72	4.58	3.42	36.35
FGO-GNSS/PDR	2.97	2.66	1.31	6.55
FGO-GNSS/PDR + Anchor	1.25	1.19	0.40	2.10

**Table 6:** Improvement rates of GNSS and FGO-GNSS/PDR + Anchor compared to the baseline in Experiment 3.

Method	RMSE (%)	MEAN (%)	STD (%)	MAX (%)
GNSS	−93%	−72%	−162%	−455%
FGO-GNSS/PDR	—	—	—	—
FGO-GNSS/PDR + Anchor	58%	55%	70%	68%

**Table 7:** PDR-only localization errors (RMSE, MEAN, STD, MAX) in Experiment 2.

Group	RMSE (m)	MEAN (m)	STD (m)	MAX (m)
1st	15.07	13.93	5.76	26.48
2nd	12.00	11.08	4.61	19.50
3rd	9.17	8.53	3.36	15.32

**Table 8:** Anchor-matching errors (in meters) across five anchors in Experiment 1.

Dataset	Anchor_1_	Anchor_2_	Anchor_3_	Anchor_4_	Anchor_5_
1st	0.19	0.13	0.22	0.32	0.11
2nd	1.27	2.54	0.61	0.17	0.70
3rd	0.56	0.46	0.46	0.74	0.30

**Table 9:** Anchor-matching performance in Experiment 1.

Dataset	RMSE (m)	MEAN (m)	STD (m)	MAX (m)
1st	0.21	0.19	0.08	0.32
2nd	1.34	1.06	0.82	2.54
3rd	0.66	0.64	0.16	0.75

**Table 10:** Positioning performance before and after anchor refinement for the 2nd dataset in Experiment 1.

Stage	RMSE (m)	MEAN (m)	STD (m)	MAX (m)
Before Refinement	1.65	1.45	0.78	3.08
After Refinement	1.38	1.14	0.79	3.21

**Table 11:** Positioning performance of the listed methods in the another-user dataset.

Method	RMSE (m)	MEAN (m)	STD (m)	MAX (m)
GNSS	6.75	5.14	4.38	41.87
FGO-GNSS/PDR	2.76	2.51	1.17	4.85
FGO-GNSS/PDR + Anchor	1.32	1.19	0.58	2.56

**Table 12:** Improvement rates of GNSS and FGO-GNSS/PDR + Anchor compared to the baseline in the another-user dataset.

Method	RMSE (%)	MEAN (%)	STD (%)	MAX (%)
GNSS	−144%	−105%	−276%	−763%
FGO-GNSS/PDR	—	—	—	—
FGO-GNSS/PDR + Anchor	52%	53%	50%	47%

**Figure A1:**
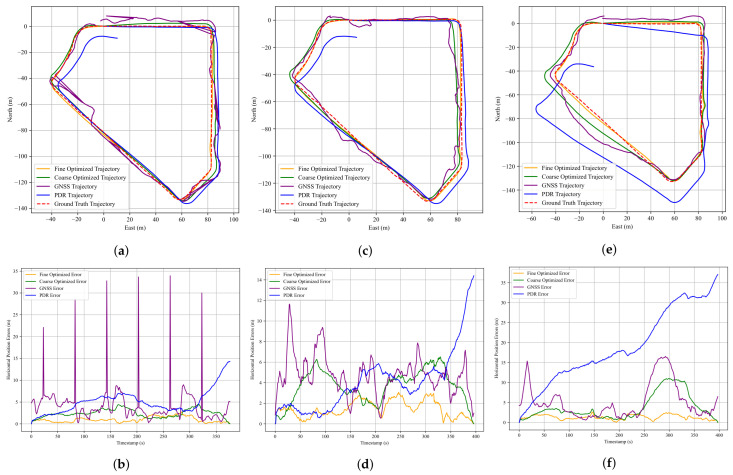
Visualizations of Experiment 1 results. (**a**,**c**,**e**) show the estimated trajectories for Datasets 1, 2, and 3, respectively. (**b**,**d**,**f**) present the corresponding horizontal position errors for the same datasets. In these plots, the red dashed line denotes the ground truth, the blue solid line indicates the PDR result, the purple solid line represents the GNSS result, the green solid line shows the FGO-GNSS/PDR result, and the orange solid line corresponds to the FGO-GNSS/PDR + Anchor result.

**Figure A2:**
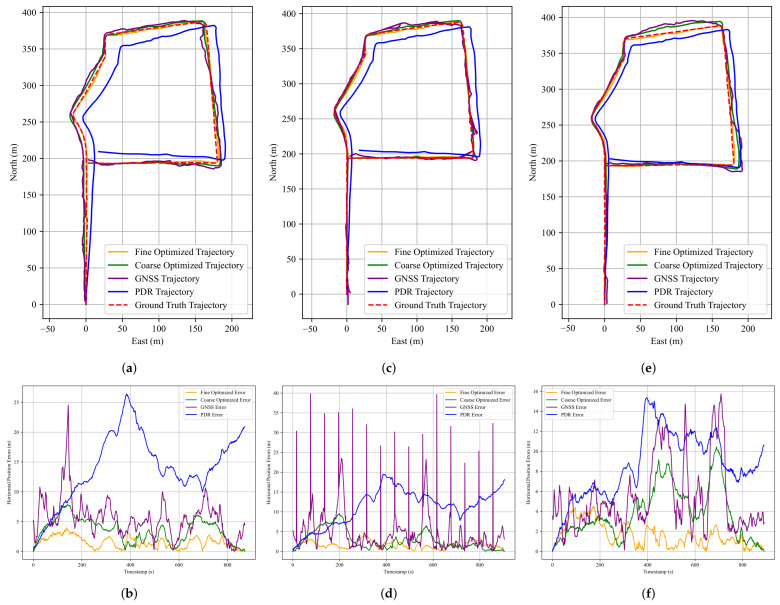
Visualizations of Experiment 2 results. (**a**,**c**,**e**) show the estimated trajectories for Datasets 1, 2, and 3, respectively. (**b**,**d**,**f**) present the corresponding horizontal position errors for the same datasets. In these plots, the red dashed line denotes the ground truth, the blue solid line indicates the PDR result, the purple solid line represents the GNSS result, the green solid line shows the FGO-GNSS/PDR result, and the orange solid line corresponds to the FGO-GNSS/PDR + Anchor result.

## Data Availability

The data presented in this study and the source code are available on request from the corresponding author due to privacy reasons.

## Appendix A

### Appendix A.1

Detailed visualizations of the results from Experiment 1 are provided in Figure A1. Subfigures (a), (c), and (e) show the trajectory plots for Datasets 1, 2, and 3, respectively. Subfigures (b), (d), and (f) illustrate the corresponding horizontal position errors for these datasets. In these plots, the red dashed line denotes the ground truth, the blue solid line indicates the PDR result, the purple solid line represents the GNSS result, the green solid line shows the FGO-GNSS/PDR result, and the orange solid line corresponds to the FGO-GNSS/PDR + Anchor result.

### Appendix A.2

Detailed visualizations of the results from Experiment 2 are provided in Figure A2. Subfigures (a), (c), and (e) show the trajectory plots for Datasets 1, 2, and 3, respectively. Subfigures (b), (d), and (f) illustrate the corresponding horizontal position errors for these datasets. In these plots, the red dashed line denotes the ground truth, the blue solid line indicates the PDR result, the purple solid line represents the GNSS result, the green solid line shows the FGO-GNSS/PDR result, and the orange solid line corresponds to the FGO-GNSS/PDR + Anchor result.
